# Proactive Screening Beliefs in Chinese High-Risk Patients of Panvascular Disease from the Perspective of Health Belief Model: A Qualitative Study

**DOI:** 10.3390/healthcare14121766

**Published:** 2026-06-18

**Authors:** Shuying Li, Xin Xu, Chenxu Huang, Yuan Yu, Yu Chen

**Affiliations:** 1 School of Nursing, Fudan University, Shanghai 200032, China; 24211170047@m.fudan.edu.cn (S.L.); 23211170034@m.fudan.edu.cn (X.X.); 2 Zhongshan Hospital, Fudan University, Shanghai 200032, China; huang.chenxu@zs-hospital.sh.cn (C.H.); yu.yuan@zs-hospital.sh.cn (Y.Y.)

**Keywords:** panvascular disease, proactive screening, health belief, qualitative study, proactive health

## Abstract

**Background**: Panvascular disease (PVD) is a systemic atherosclerotic condition that poses a substantial threat to global health. Despite the recognized importance of early proactive screening, proactive screening beliefs among high-risk populations are poorly understood. **Objective**: To explore the proactive screening beliefs among Chinese high-risk patients for PVD based on the Health Belief Model (HBM), so as to provide evidence for developing targeted nursing intervention strategies and health policies. **Methods**: A descriptive qualitative study was conducted. Employing a purposive sampling strategy with maximum variation, participants at elevated risk for PVD were recruited from a tertiary hospital in Shanghai between October and December 2025 to conduct semi-structured interviews. Data saturation guided sample size (*n* = 22; 14 male, 8 female; mean age 62.68 years). Data were analyzed using qualitative content analysis. **Results**: Five main themes were extracted: multifaceted perceptions of susceptibility, multidimensional fear of severity, positive attitudes toward the benefits of proactive screening, multiple perceived barriers to proactive screening, and significant differences in self-efficacy for proactive screening. **Conclusions**: The proactive screening beliefs in Chinese high-risk patients of PVD were deeply embedded in local cultural values and healthcare realities. Tailored health education, age-friendly service optimization, and stratified intervention strategies are urgently needed to reduce screening barriers and improve population-wide proactive screening beliefs.

## 1. Introduction

Panvascular disease (PVD) refers to a group of systemic vascular disorders characterized by atherosclerosis as a common pathological feature, often involving multiple vascular territories, such as the heart, brain, and peripheral vessels [1,2,3,4,5]. With the widespread prevalence of risk factors such as an unhealthy diet, physical inactivity, and smoking, the incidence of hypertension, diabetes mellitus, and dyslipidemia continues to rise, contributing to an increasing burden of PVD [6,7]. PVD has been recognized as a leading cause of mortality and disability worldwide [8]. Compared with single-vessel disease, PVD carries a higher risk of adverse cardiovascular events as the number of affected vascular beds increases [9,10], with approximately 10% of single-vessel patients progressing to multivessel involvement within three years [11]. Characterized by its systemic nature, progressive course, and clinical severity, PVD has emerged as a major public health threat.

In response to this serious situation, early screening and intervention are widely recognized as key strategies for the prevention and management of PVD [12,13]. Relevant guidelines recommend systematic risk assessment for individuals aged ≥40 years with major vascular risk factors or established single-territory atherosclerotic cardiovascular disease, integrating them into a panvascular-centered screening pathway [12]. Currently recommended screening modalities include carotid ultrasound, cardiac computed tomography, the ankle-brachial index, and fundus photography [14]. However, public health awareness often remains confined to an organ-specific, “treat-the-symptom” model, with limited understanding of PVD as a novel, systemic concept, resulting in low initiative and adherence. Consequently, many at-risk individuals missed opportunities for timely intervention and the disease burden continues to rise.

Despite the recognized importance of early screening, existing studies [14,15] have predominantly focused on optimizing screening techniques and clinical indicators, with little exploration of individual cognitive and behavioral mechanisms. In particular, there is a lack of qualitative evidence exploring the psychological and social determinants of proactive screening behavior among high-risk populations. Understanding why individuals do or do not engage in proactive screening requires an in-depth exploration of their beliefs, fears, perceived benefits, barriers, and self-efficacy—factors that quantitative surveys can only capture superficially.

The Health Belief Model (HBM), as a classical theoretical framework for explaining individual health behavior decision-making, links “beliefs” to health behaviors [16]. The HBM posits that an individual’s beliefs, including perceived susceptibility to a disease, perceived severity of the disease, perceived benefits of the recommended health action, perceived barriers to taking that action, and self-efficacy, directly influence their health decisions and behavioral choices [16]. The HBM has been widely applied in the prevention and management of chronic diseases with demonstrated effectiveness, and has also shown distinct advantages in research related to disease screening [17,18].

China provides a particularly relevant context. As of 2025, over 310 million people are aged ≥60 years, while the total number of cardiovascular disease cases has reached 330 million [19]. Despite basic medical insurance covering over 95% of the population, out-of-pocket spending still accounted for 27.3% of total health expenditure [20]. In large tertiary hospitals, patients frequently face the so-called “three longs and one short” phenomenon, namely long registration, long waiting, long medicine collection, and short consultation time, leading to great discontent and avoidance of care [21]. Additionally, only 30.45% of older adults have adequate digital health literacy [22]. Such barriers disproportionately affect preventive care use.

A qualitative approach is ideally suited to capture these culturally embedded factors. Unlike quantitative surveys, which can only measure pre-defined variables and may miss unexpected influences, in-depth semi-structured interviews allow participants to articulate their beliefs and decision-making processes in their own words. This method is particularly valuable for exploring sensitive topics and for uncovering the complex interplay among social, cultural, and psychological factors that shape screening behavior—a level of understanding that cannot be achieved through closed-ended questionnaires.

Therefore, this study aimed to conduct a qualitative study to explore beliefs regarding proactive PVD screening among high-risk individuals using the HBM as an analytical lens. Specifically, we sought to describe how high-risk patients perceive their susceptibility to PVD and the severity of the disease; identify their perceived benefits and barriers to proactive screening; and examine their self-efficacy in navigating screening processes. The findings are intended to provide empirical evidence for developing targeted nursing interventions and effective screening promotion strategies.

## 2. Subjects and Methods

### 2.1. Study Design

This was a descriptive qualitative study designed to explore the cognitive characteristics of beliefs toward proactive screening among high-risk patients with PVD. The study adhered to the Consolidated Criteria for Reporting Qualitative Research (COREQ) to ensure methodological rigor and transparency [17]. Ethical approval was obtained from the Ethics Committee of the School of Nursing, Fudan University (IRB# 2025-07-7).

### 2.2. Setting and Participants

This study employed purposive sampling to recruit participants. Participants were recruited between October and December 2025 from a tertiary hospital in Shanghai, China. This hospital is a well-known general hospital with high patient consultation and hospitalization rates and rich disease diversity, thereby ensuring the clinical representativeness of the study sample. The first author (S.L.) is completing a clinical internship at this hospital, enabling her to screen electronic inpatient medical records and consult with attending physicians to confirm each patient’s eligibility based on documented diagnoses, risk factors, and comorbidities.

Inclusion criteria were as follows: (1) presence of major vascular risk factors (e.g., family history of premature cardiovascular disease, familial hypercholesterolemia, smoking, hypertension, diabetes, hyperlipidemia, obesity) or confirmed single-territory atherosclerotic cardiovascular disease (e.g., acute coronary syndrome, history of myocardial infarction, angina pectoris, coronary or other vascular revascularization, stroke, transient ischemic attack, peripheral artery disease, etc.) [14]; (2) a conscious state with normal communication and comprehension abilities; and (3) voluntary participation with informed consent. Exclusion criteria were: (1) cognitive or mental disorders and (2) concomitant severe diseases such as malignancy.

A total of 32 patients were recruited. Of these, 24 met the inclusion criteria and agreed to participate. Two of these served as pilot tests, while the remaining 22 were for formal interviews (14 male, 8 female; age range 44–77 years). The reasons for exclusion were: cognitive impairment (*n* = 2), malignancy (*n* = 2), and refusal (*n* = 4). Maximum variation sampling was used to ensure diversity in age, education, living situation, and risk factors for PVD. The sample size was determined based on the principle of data saturation. Specifically, recruitment was terminated when no new codes or themes emerged across three consecutive interviews [23]. Saturation was reached after the 19th interview, with interviews 20–22 confirming no additional themes.

### 2.3. Instruments

This study employed a semi-structured interview approach, developed based on the researchers’ expertise and a review of the relevant literature. The interviewer was a female graduate student in nursing who had received systematic training in qualitative research and had mastered the principles, techniques, and procedures of qualitative interviewing. The researcher was also familiar with the relevant research content. The semi-structured interview guide was developed based on the HBM, a literature review and team discussions. An expert panel of three members (two nursing professors with cardiovascular expertise and one qualitative methodologist) reviewed the guide for relevance and comprehensiveness. A pilot test was conducted with two participants (not included in the main study) to confirm the clarity and validity of the questions. Based on feedback from the pilot, ambiguous questions were reworded and prompts for each HBM construct were added. The final version of the interview outline (Supplementary File S1) consisted of 11 main questions. A mapping table linking each question to HBM constructs is provided in Supplementary File S1.

### 2.4. Data Collection

This study collected data through one-on-one, face-to-face semi-structured interviews in a private room to ensure a comfortable environment. All interviews were arranged at a time convenient for participants. Each interview lasted between 30 and 60 min, and was audio-recorded with the written informed consent of all participants. Field notes documented nonverbal behaviors. Given that PVD is a relatively new concept for the general public, the researcher provided a standardized definition to ensure participants understood the condition before discussing screening beliefs. Throughout the interview, the researcher maintained a neutral stance, avoiding the imposition of personal opinions or leading questions. Open-ended probing questions—such as “Could you describe in more detail what you were thinking at that time?” and “Were there any other factors that influenced your decision?”—were used to encourage participants to fully share their perceptions, attitudes, and experiences related to proactive screening beliefs for PVD. All interviews were conducted in Mandarin Chinese. Audio recordings were transcribed verbatim in Chinese within 24 h. The transcribed texts were then cross-checked with the original recordings. If any questions or ambiguities arose, the researcher returned to the participant for verification to ensure the accuracy and completeness of the data. All identifiable information of participants was replaced with anonymous codes during transcription to protect privacy. All data files were stored on an encrypted, password-protected computer, and only the research team could access the data.

### 2.5. Data Analysis

Transcripts were imported into NVivo 12.0 software (QSR International Pty Ltd., Doncaster, VIC, Australia), which fully supports Chinese text. Qualitative content analysis followed a hybrid inductive-deductive approach. The primary researcher (S.L.) independently read all transcripts, identified meaning units, and generated initial codes inductively without imposing a pre-existing framework. Similar codes were then grouped into subcategories, then into subthemes based on emergent patterns. Subsequently, these subthemes were deductively mapped onto the HBM constructs. Codes that did not align with HBM were preserved as separate context rather than being forced into inappropriate constructs. A detailed codebook was maintained throughout the analysis. Analytical memos were written during coding. The second researcher (X.X.) reviewed the interview transcripts and verified the codes and themes developed by the first researcher (S.L.). Any disagreements were discussed between the two researchers until consensus was reached. Finally, all authors convened in group meetings to collectively discuss the accuracy and appropriateness of the themes and subthemes until full consensus was achieved. The processing and encoding of raw data were conducted within a Chinese context to avoid semantic loss or bias due to translation. This ensured that the generated codes and themes remained grounded in the original linguistic and cultural background. For quotations selected for publication, a forward–backward translation method was used: a bilingual researcher translated the texts from Chinese to English, and a second independent bilingual translator back-translated them into Chinese. Discrepancies were resolved by consensus. To ensure translation quality, the process involved manual translation followed by verification and linguistic refinement using AI-assisted translation tools.

### 2.6. Research Trustworthiness

The trustworthiness of this study was established by systematically addressing the four criteria proposed by Lincoln and Guba [24]: credibility, transferability, dependability, and confirmability. Credibility was enhanced via member checking (five participants confirmed that the interpreted findings accurately reflected their views) and peer debriefing, with the research team holding regular meetings to review coding and thematic development. Transferability was supported by describing the study context, participant characteristics, and the Chinese healthcare setting, helping readers assess applicability. Dependability was maintained through a comprehensive audit trail documenting research decisions, coding revisions, and analytical memos. Audio recordings were transcribed by two researchers (S.L. and X.X.) and checked by another experienced in qualitative studies, who confirmed the analysis. To ensure confirmability, all findings were grounded in participants’ verbatim quotations. Furthermore, the researcher engaged in bracketing by deliberately suspending preconceived assumptions, personal values, and prior theoretical knowledge, remaining open to the participants’ experiences. All coding decisions were reviewed by the research team to minimize bias.

### 2.7. Reflexivity Statement

The first author (S.L.) conducted all interviews. S.L. is a female graduate student in nursing with training in qualitative research methods. At the time of the study, she was completing a clinical internship at the recruitment hospital and had provided direct care to some potential participants. This prior relationship could have influenced participants’ responses or the researcher’s interpretation. To minimize psychological burden and potential bias when discussing sensitive topics, the following measures were implemented: (1) All interviews were conducted in a private room away from the clinical unit and outside the interviewer’s clinical shifts, ensuring a non-clinical, comfortable environment. (2) Participants were explicitly informed that they could skip any question or terminate the interview at any time without affecting their medical care. (3) The researcher used open-ended, non-leading questions and avoided probing on particularly distressing topics unless the participant initiated. (4) If a participant showed signs of distress, the interview was paused, and the participant was offered emotional support or the option to stop. No participant required early termination.

## 3. Results

In this study, a total of 22 patients were interviewed. All personal identifiers were removed and replaced with anonymous codes (P1–P22). Among them, there were 14 (63.6%) male and 8 (36.4%) female patients, with ages ranging from 44 to 77 (62.68 ± 10.11) years, covering middle-aged to older adult populations. Their height was (165.09 ± 5.82) cm, weight was (69.86 ± 8.44) kg, and body mass index (BMI) was (25.52 ± 1.43) kg/m^2^. Detailed information regarding these participants, including age, educational level, living situation, and relevant medical history, is summarized and presented in Table 1. Analysis of the interview data produced 504 unique codes. Similar codes were grouped into 80 subcategories, then into 19 subthemes, and finally into 5 themes (Table 2).

### 3.1. Theme 1: Multifaceted Perceptions of Susceptibility

#### 3.1.1. Unhealthy Lifestyle Habits

Participants generally viewed long-term unhealthy lifestyle habits as the core risk factors for PVD. Smoking, alcohol consumption, high-fat and high-sugar dietary preferences, staying up late, sedentary lifestyles and physical inactivity were frequently mentioned as harmful daily habits. Most participants believed these cumulative daily behavioral patterns gradually damaged vascular condition and elevated disease vulnerability.

*“Long-term unhealthy habits such as smoking, alcohol consumption, a diet high in fatty meats and offal, and late-night sleep deprivation directly impair vascular health.”*

(P1)

*“Prolonged sedentary work, such as in accounting, leads to chronic physical inactivity, impaired blood circulation, and inevitably exerts negative effects on vascular health.”*

(P4)

*“Unhealthy dietary habits, including a preference for high-fat and high-sugar foods, coupled with irritability and fatigue, predispose individuals to these conditions.”*

(P7)

#### 3.1.2. Age and Underlying Diseases

Most middle-aged and older participants associated aging with increased vascular disease susceptibility, perceiving physiological function decline with age as an inherent vulnerability to vascular lesions. Participants also explicitly identified hypertension, hyperglycemia, and hyperlipidemia as direct risk factors. They acknowledged that these chronic underlying conditions are closely related to the occurrence and progression of panvascular lesions. Additionally, some participants with established single-territory cardiovascular conditions also gradually realized the systemic nature of vascular impairment beyond single-lesion sites.

*“With advancing age, weakened immunity and declining organ functions create vulnerabilities, allowing unhealthy habits to take hold and diseases to develop.”*

(P15)

*“I believe individuals with hypertension, diabetes, and hyperlipidemia—these ‘three highs’—are more susceptible to panvascular disease.”*

(P5)

*“High blood lipids, glucose, and pressure all damage vessels: lipids cause fat accumulation and hardening, pressure makes vessels brittle, and high glucose causes insidious harm.”*

(P8)

*“I have coronary heart disease, which may involve systemic vascular problems, though plaques elsewhere are mild.”*

(P6)

#### 3.1.3. Heredity and Family Aggregation

Family health history was widely perceived by participants as an important determinant of personal disease risk. Participants often formed subjective risk judgments based on multigenerational family disease patterns, tending to perceive their own susceptibility as inevitable due to genetic aggregation.

*“I have bad genes—my father had a stroke and family members have diabetes, so I’m likely affected too. It’s hereditary.”*

(P2)

*“I have had such concerns, after all, both my father and grandmother had hypertension and coronary heart disease (CHD).”*

(P4)

*“Hypertension runs in our family for generations. My father had hypertension, diabetes, and CHD, and a cousin had a stroke. I knew from retirement that I couldn’t escape these diseases.”*

(P9)

#### 3.1.4. Stress and Emotion

Participants acknowledged that long-term mental stress, emotional fluctuations and negative mood states were closely linked to vascular health. They perceived persistent life pressure, irritability and emotional suppression as unfavorable factors affecting physical and vascular status. Many participants reported that ongoing family care responsibilities, work pressure and emotional strain placed a continuous burden on physical well-being.

*“A poor mood and stress can affect blood pressure and emotional state, which is certainly detrimental to cardiovascular health.”*

(P1)

*“Emotional impact is substantial, leading to irritability and heightened stress, both of which are harmful to blood vessels. I sometimes get angry and am under a lot of stress, as I have to work, support elderly family members, and ensure my children’s education—these factors may also contribute to this condition.”*

(P3)

### 3.2. Theme 2: Multidimensional Fear of Severity

#### 3.2.1. Loss of Physical Function and Dignity

When reflecting on the consequences of PVD, participants were most concerned about physical dysfunction, paralysis and impaired self-care ability. Such outcomes were perceived to severely diminish quality of life and personal autonomy. Some participants even expressed extreme resistance to long-term bedridden status, viewing loss of independence as unacceptable.

*“It could affect my hands or feet, or leave me partially paralyzed—any of that would make life difficult.”*

(P11)

*“As a cancer patient with an ostomy, being bedridden would make cleaning the bag extremely difficult.”*

(P5)

*“I feel that if I ended up with lasting effects, I’d rather be dead.”*

(P1)

*“I’d rather die of a heart attack than just lie there unable to move. If it came to that, dying would be the cleaner way out—no more dragging things along.”*

(P20)

#### 3.2.2. Burden of Family Care

Most participants regarded potential illness as a family event rather than merely a personal health issue. They were deeply worried that severe illness would impose heavy caregiving responsibilities on family members. They expressed guilt about shifting family roles from being a supporter to a person requiring long-term care.

*“If the illness is severe, a family member must stay and care for you, taking over your work and bearing an extra burden.”*

(P8)

*“No one feels at ease when a family member is sick. My wife even wakes up at night to check my breathing. I can’t do heavy work, so chores fall on her.”*

(P16)

#### 3.2.3. Economic Crisis

Medical expenditure, long-term medication, rehabilitation and caregiving costs were prominent concerns among participants. Even with medical insurance, out-of-pocket expenses were perceived as a notable financial burden for ordinary families. Middle-aged participants who were the main household earners also worried that illness would interrupt work and reduce family income.

*“Caregivers, medications, check-ups, and rehabilitation all cost money.”*

(P3)

*“For instance, the surgery I underwent this time cost around 40,000 to 50,000 yuan. After health insurance reimbursement, I still had to pay over 10,000 yuan out-of-pocket.”*

(P10)

*“If it happened to someone younger—like me—there’s no way I could go back to work, and that would cut off the income. For people like us who work for ourselves, losing that source of income is a huge blow.”*

(P8)

#### 3.2.4. Psychological Burden and Lifestyle Restrictions

Participants reported that severe vascular disease would bring sustained psychological pressure, anxiety and pessimistic emotions. They also anticipated unavoidable changes to personal hobbies, daily routines and lifestyle freedom. Many participants also expressed persistent anxiety about disease recurrence, creating long-term psychological unease even after clinical recovery.

*“The psychological pressure is definitely substantial. After falling ill, it’s nearly impossible to maintain an optimistic outlook for most.”*

(P12)

*“I used to do weightlifting, swimming, billiards, and go to the gym often. From now on, I probably won’t be able to handle strenuous activities.”*

(P11)

*“There’s always a knot in my heart. I’m constantly haunted by concerns about recurrence or worsening.”*

(P10)

### 3.3. Theme 3: Positive Attitude Toward the Benefits of Proactive Screening

#### 3.3.1. Early Warning and Clinical Benefits

Participants recognized that proactive screening enabled early detection and timely intervention of hidden vascular problems. Early identification was believed to simplify treatment, control disease progression, and avoid complex surgical interventions.

*“Proactive screening helps us detect problems earlier and intervene in time, keeping blood lipids and pressure under control and avoiding surgery.”*

(P16)

*“My vessels are over 70% blocked. If screened earlier when blockage was under 50%, I could have avoided a stent. Earlier detection means simpler treatment.”*

(P5)

#### 3.3.2. Psychological Security and Family Responsibility

Proactive screening was viewed as an effective way to relieve health anxiety and obtain psychological reassurance about hidden physical risks. Meanwhile, participants regarded personal health maintenance and active screening as a basic responsibility to their families.

*“It helps ease the worry about whether something might be wrong with your health.”*

(P7)

*“Since I’m not entirely sure about some things, I feel it’s better to do more tests. Just feeling okay doesn’t necessarily mean you’re truly healthy.”*

(P12)

*“Proactive screening is about taking responsibility for myself and for my family. My health is a source of happiness for my family.”*

(P3)

#### 3.3.3. Trigger for Health Behavior Change

Participants noted that objective screening indicators could directly alert individuals to unrecognized health risks, breaking subjective complacency and prompting lifestyle adjustments. For patients with prior cardiovascular interventions, regular screening was seen as necessary to monitor stent patency and long-term disease status.

*“Not everyone has symptoms. My physical exam found calcified plaques, so I became vigilant and started regular check-ups.”*

(P3)

*“Test results help target habit changes, like reducing greasy food. Sometimes you need those indicators to drive change.”*

(P14)

### 3.4. Theme 4: Multiple Perceived Barriers to Proactive Screening

#### 3.4.1. Asymptomatic Neglect and Cognitive Blind Spots

Most participants held the view that a medical examination was unnecessary without obvious physical discomfort. They tended to equate the absence of symptoms with good health and overlooked the asymptomatic characteristics of vascular lesions. Many participants also lacked systematic knowledge of PVD and relevant screening procedures, with limited access to professional health education.

*“Only sick people get proactive checks. If I feel fine, I won’t get tested. Most people still just treat the symptoms as they arise—headaches get head treatment, foot pain gets foot treatment.”*

(P8)

*“I eat well and sleep well. I don’t have any issues, so I don’t need to get checked.”*

(P17)

*“I don’t know what screening tests are available or how to get them. No one has explained the process.”*

(P6)

#### 3.4.2. Economic Burden and Value Trade-Off

Older and economically constrained participants were reluctant to spend on preventive screening. They tended to question the value of medical expenditure if screening results appeared normal.

*“Older or low-income individuals worry about spending on tests that show nothing wrong.”*

(P3)

*“Elderly individuals often lack a proper understanding of economic benefits, believing that spending money on medical care is not worthwhile and that it should not be wasted. They are accustomed to frugality. Sometimes, even when no abnormalities are detected, they may feel that the money was spent unnecessarily.”*

(P15)

#### 3.4.3. Cumbersome and Inefficient Processes

Long waiting times and repeated queuing for registration, payment and examinations were commonly complaints by participants. Complicated hospital procedures reduced their willingness to attend proactive screening. Older participants reported particular difficulties in adapting to complex in-hospital workflows and crowded medical environments.

*“Large hospitals have long queues—registering, seeing the doctor, paying, and testing all require waiting. Repeated queuing is frustrating.”*

(P11)

*“Older adults face difficulties: they can’t use online appointments, must queue at windows for long periods, and may have mobility issues. Crowded hospitals risk being jostled.”*

(P3)

#### 3.4.4. Digital Divide and Technical Barriers

Smartphone-based appointments, mobile payment and self-service machine operations posed obvious challenges for older participants with limited digital literacy. Many felt unfamiliar and frustrated with digitized medical services.

*“Many elderly people find smartphones difficult. Registering and making appointments on phones is troublesome for them.”*

(P8)

*“Nowadays, most operations are done on phones or computers, which can be inconvenient for us older folks. We don’t understand how to use machines for registration, payment, or similar tasks.”*

(P19)

#### 3.4.5. Disease-Related Fear and Fatalistic Beliefs

Some participants avoided screening due to fear of detecting unexpected severe illness, which created psychological anxiety about examination outcomes. A number of older participants held fatalistic views regarding health and the inevitability of disease. In addition, some individuals avoided regular check-ups to evade social judgment of “fear of death”.

*“I usually avoid check-ups out of fear. Finding something wrong would weigh on my mind. Normal results relieve me; abnormal results keep me awake.”*

(P20)

*“Life is predetermined by heaven. Health cannot be self-determined. The fortunate are protected.”*

(P19)

*“Some people go for check-ups all the time—those are the ones who are afraid to die. I don’t want to be like that. I don’t go for check-ups. I’m not afraid.”*

(P17)

### 3.5. Theme 5: Significant Differences in Self-Efficacy for Proactive Screening

#### 3.5.1. Rich Experience Fosters Independent Confidence

Participants with long-term hospital visiting experience were familiar with hospital layout, examination procedures, and department distribution, and expressed high confidence in completing screening independently.

*“I’ve been through so many check-ups that I’m experienced now. I don’t worry anymore.”*

(P15)

*“I go alone—I know the hospital and test order well. Frequent visits teach you the process.”*

(P1)

#### 3.5.2. Tools and Resources Enable Smooth Navigation

Other participants reported that clear, step-by-step instructions on the examination order form enabled them to navigate the required tests with confidence. In addition, some participants actively used online platforms and social media to acquire screening knowledge, examination preparation guidance, and result interpretation information, which helped them arrange medical visits more smoothly.

*“The examination form clearly shows the time and location for each test. Now I can also look up my results online. For instance, if my blood lipids are high, I can search for the causes and treatment targets myself.”*

(P15)

*“I searched online beforehand. Social media like WeChat and Xiaohongshu are helpful for learning about screening.”*

(P16)

#### 3.5.3. High Dependency Undermines Action Confidence

Older participants, those unfamiliar with digital medical services, and individuals who habitually relied on others depended heavily on family members for hospital accompaniment and assistance. Without family support, they tended to delay or give up screening arrangements.

*“I need my daughter to come with me every time I see a doctor. At my age, I don’t know how to use mobile payments or make online appointments.”*

(P2)

*“A family member should accompany me to hospital tests—it gives me peace of mind.”*

(P9)

*“When going alone, I procrastinate—a blood test might be delayed for weeks or months. Someone supervising helps me go on time.”*

(P14)

## 4. Discussion

### 4.1. Proactive Screening Beliefs in High-Risk Patients of PVD

This study revealed a distinctive contribution to the literature, namely a detailed qualitative mapping of each HBM construct onto the lived experiences of individuals at risk for PVD. Unlike previous studies applying the HBM to single-vessel disease management [25], our findings captured the systemic, multiterritorial nature of PVD, which amplifies both perceived threat and decisional complexity.

Participants’ perceptions of susceptibility were multidimensional, encompassing unhealthy lifestyle patterns, aging, underlying chronic conditions, hereditary, and psychological stress. Although participants recognized conventional vascular risk factors, a prominent cognition–belief disconnect persisted: most individuals acknowledged disease vulnerability but ignored the asymptomatic and progressive nature of systemic atherosclerosis. This reflected the long-standing Chinese health perception that prioritizes treating overt illness over preventing latent disease [26]. Middle-aged and older participants tended to attribute vascular degeneration to irreversible aging and familial genetic fate, showing passive acceptance rather than proactive prevention. Influenced by Eastern emotional restraint norms [27], participants often suppressed chronic stress and negative emotions, with cumulative vascular damage constituting a notable risk.

When evaluating disease severity, participants’ concerns extended far beyond physical dysfunction to include family caregiving burdens, economic pressure, psychological distress, and lifestyle constraints. Different from the individual-oriented values in Western populations, Chinese respondents exhibited typical family-centred cognition [28]. Fear of physical disability was largely followed by the worry of becoming a financial and care burden on spouses and offspring, reflecting collectivism and intergenerational responsibility. Anxiety over medical costs, out-of-pocket expenses and income interruption further amplified perceived severity, particularly among retired elderly and middle-aged breadwinners in the context of China’s current medical insurance and pension system [29,30]. Studies have shown that chronic diseases significantly increase the risk of catastrophic health expenditure in elderly Chinese households, with basic medical insurance failing to fully alleviate this burden [31,32]. Over 80% of Chinese older adults rely on informal care, imposing substantial psychological and economic costs on family caregivers [33].

In terms of perceived benefits, participants affirmed the clinical value of early warning and timely intervention, and valued screening for psychological reassurance, family responsibility, and lifestyle correction guided by objective indicators. Beyond clinical gains, the psychological benefit of screening reflects a cognitive shift from uncertainty to perceived control. Haslam-Larmer et al. [34] demonstrated that patients actively sought imaging to resolve diagnostic uncertainty and gain a sense of control over their condition. In our study, this mechanism was amplified by family responsibility. As Yue et al. [35] found, chronic disease management in Chinese society was embedded in family obligations. Moreover, objective indicators break the “no symptoms, no disease” mindset, providing actionable feedback loops that drive lifestyle change [36]. For patients with established single-territory disease, regular screening was also recognised as necessary to monitor disease progression, demonstrating rational long-term disease management awareness.

Multiple perceived barriers mirrored China’s healthcare delivery and aging challenges. Asymptomatic neglect, economic concerns, cumbersome hospital workflows, and the digital divide collectively hinder screening participation. Culturally rooted fatalistic beliefs and social stigma, which view frequent check-ups as an excessive fear of death, further deter proactive engagement [8]. These barriers are shaped by the centralization of high-quality medical resources, limited age-friendly services, and traditional frugality. Hukou-based inequalities are associated with earlier screening among urban residents, reflecting structural differences affecting rural populations [37]. Cost concerns, amplified by low income as the most common screening barrier [38], together with fatalism [39] reflect traditional beliefs associating disease with fate, reducing perceived controllability. Limited digital health literacy disproportionately affects preventive service utilisation [40]. Collectively, resource constraints, digital disparities, and fatalistic worldviews not only perpetuate screening avoidance but also highlight that low screening uptake is not merely an individual knowledge deficit but a product of intersecting structural and cultural forces that demand multi-level, context-sensitive interventions.

Screening self-efficacy showed substantial heterogeneity stratified by healthcare experience, digital literacy, and family support. Specifically, participants who had previously received regular healthcare services reported significantly higher confidence in completing screening procedures correctly. This finding aligns with existing research that links accumulated positive healthcare interactions to greater perceived control over preventive health behaviors [41]. Meanwhile, patients with social media proficiency maintained high independent screening confidence, consistent with Lu [42], who confirmed a positive correlation between mHealth use and self-efficacy among older Chinese adults. Conversely, older patients with low digital skills and heavy family care reliance lacked confidence without accompaniment. Chen et al. [43] identified strong needs for medical visit accompaniment services in this population. With only 30.45% of Chinese older adults possessing adequate digital health literacy [22], structural inequalities in eHealth access further widen the self-efficacy gap, reinforcing dependence on family assistance.

These findings directly inform the practical recommendations presented in the following section.

### 4.2. Implications for Policy and Practice

The findings of this study carry practical implications for clinical nursing practice, community health management, and national public health policy formulation. In terms of clinical nursing, standard education should replace uniform content delivery with personalized risk communication. Clinical nurses should adopt visualized vascular lesion data and dynamic health monitoring results to convert abstract long-term vascular risks into immediate and perceptible threats, so as to correct the misconception of asymptomatic neglect. Hospitals need to optimize age-friendly medical services by retaining manual registration and payment windows, arranging volunteer guidance for self-service equipment and simplifying examination procedures, thereby reducing procedural barriers for older and low-digital-literacy populations. Moreover, targeted health skill training and peer sharing activities might be used to effectively improve screening self-efficacy, and family-collaborative intervention strategies should be formulated for highly dependent older patients to enhance their screening initiative.

For community health practice, grassroots medical institutions should take the lead in carrying out localized PVD health education rooted in Chinese family culture, popularizing the harm of asymptomatic atherosclerotic lesions and breaking the traditional mindset of “treating illness rather than preventing disease”. Integrated PVD screening pathways should be established for high-risk groups, sinking screening resources into communities and primary care centres to reduce the time and economic cost of seeking medical treatment in tertiary hospitals. For vulnerable groups, including rural older adults, low-income residents, and populations with lower educational levels, community-based home health assessments and free or subsidized screening programmes are recommended to reduce the social disparities in screening participation.

In terms of public health policy, health administrative departments should prioritize bridging the geriatric digital divide in smart healthcare, establishing unified service standards for age-friendly medical services, and requiring medical institutions to set up special manual service windows and on-site guidance posts for older adults. It is also necessary to incorporate PVD early screening into chronic disease prevention and control priority projects, formulate hierarchical screening strategies based on age, risk factors, and socioeconomic status, and strengthen national public education campaigns to weaken fatalistic health beliefs and the social stigma associated with physical examination. Furthermore, long-term multi-dimensional support mechanisms should be developed, including optimization of medical insurance policy and guidance for preventive health investment, to reduce the economic burden of proactive screening for high-risk populations.

### 4.3. Strengths and Limitations

This study has several key strengths. First, the use of a qualitative approach allowed for an in-depth exploration of high-risk individuals’ lived experiences and subjective beliefs regarding proactive screening for PVD, filling a gap left by quantitative studies that often overlook psychosocial factors. Second, the analysis grounded in the HBM provided a robust theoretical framework for systematically examining the drivers of and barriers to screening intentions, thereby enhancing the theoretical rigor of the study. Third, the sample encompassed diverse subgroups, including older adults and those with limited technological resources, ensuring that the voices of vulnerable populations were adequately represented and thus extending the applicability of the findings to disadvantaged groups. Fourth, rigorous quality control measures were implemented to ensure the credibility and dependability of the results.

Despite these strengths, several significant limitations should be acknowledged. First, purposive sampling was used, so findings reflect the beliefs of selected inpatients and may not capture the full range of views. The sample was from a single tertiary hospital in Shanghai and consisted of inpatients, limiting transferability to community-dwelling older adults. Future community-based studies are needed. Second, the sample included patients with established single-territory cardiovascular disease. Some narratives may reflect secondary prevention rather than primary screening beliefs. This conceptual heterogeneity should be considered. Future research is needed to compare primary and secondary prevention populations to further clarify these distinct perspectives. Third, the HBM construct “cues to action” was not analyzed as a separate theme because our study focused on cognitive beliefs. Future research should examine cues to action in PVD screening. Finally, the sample had a higher proportion of males, reflecting a higher prevalence of risk factors in men, which may introduce selection bias.

## 5. Conclusions

From the perspective of the HBM, this study provides an in-depth analysis of the core components and underlying mechanisms of proactive screening beliefs among high-risk patients for PVD. It demonstrated that individuals’ screening decisions resulted from the dynamic interplay of multidimensional beliefs, including perceived susceptibility, perceived severity, perceived benefits, perceived barriers, and self-efficacy, thereby offering qualitative empirical evidence to support the development of targeted intervention strategies. The findings not only enrich theoretical research in the field of proactive screening for PVD but also provide practical guidance for clinical practice and public health policy. These results can be translated into feasible measures to optimize individualized health education, simplify cumbersome medical procedures, improve age-friendly medical services, advance community-based screening outreach, and relieve financial pressure and digital divide barriers, so as to refine nursing practice and optimize population health intervention strategies for PVD high-risk groups.

## Figures and Tables

**Table 1:** General information of participants (*n* = 22).

ID	Gender	Age (Years)	BMI (kg/m^2^)	Educational Level	Employment	Domicile	Roommates	Major Vascular Risk Factors	Diagnosed Atherosclerotic Cardiovascular Disease
P1	Male	67	25.5	Junior College	Retirement	Urban	Spouse	Hypertension, Diabetes Mellitus, Hyperlipidemia	Peripheral Arterial Disease
P2	Male	72	25.4	Junior College	Retirement	Urban	Spouse, Children	Hypertension, Diabetes Mellitus, Hyperlipidemia	Peripheral Arterial Disease
P3	Female	44	22.7	Junior College	Employed	Urban	Spouse, Children	Family History of Cardiovascular Disease, Hyperlipidemia, Smoking	Peripheral Arterial Disease
P4	Male	52	27.0	Undergraduate	Employed	Urban	Spouse, Children	Family History of Cardiovascular Disease, Familial Hypercholesterolemia, Hypertension, Diabetes Mellitus, Hyperlipidemia, Smoking	Peripheral Arterial Disease
P5	Male	74	25.0	Senior High School	Retirement	Rural	Spouse	Family History of Cardiovascular Disease, Hypertension	Peripheral Arterial Disease
P6	Female	57	24.8	Undergraduate	Employed	Urban	Spouse	Hypertension, Hyperlipidemia	/
P7	Male	62	26.0	Junior College	Retirement	Urban	Spouse	Hypertension	/
P8	Female	53	25.1	Junior College	Employed	Urban	Spouse, Children	Hypertension	Stroke
P9	Male	68	26.2	Senior High School	Retirement	Urban	Spouse, Children	Family History of Cardiovascular Disease, Familial Hypercholesterolemia, Hypertension, Diabetes Mellitus, Hyperlipidemia	Stroke
P10	Male	70	24.9	Senior High School	Retirement	Rural	Live alone	Hypertension, Smoking	Stroke
P11	Male	60	26.7	Junior College	Retirement	Urban	Spouse	Family History of Cardiovascular Disease, Hypertension, Diabetes, Smoking	Coronary Artery Revascularization
P12	Male	44	27.1	Junior College	Employed	Urban	Spouse, Children	Family History of Cardiovascular Disease, Hypertension	Coronary Artery Revascularization
P13	Female	73	22.9	Primary School	Peasant	Rural	Children	Hypertension, Hyperlipidemia	Stroke
P14	Male	49	28.4	Junior College	Employed	Urban	Spouse	Family History of Cardiovascular Disease, Familial Hypercholesterolemia, Hypertension, Diabetes Mellitus, Hyperlipidemia, Smoking	Coronary Artery Revascularization
P15	Female	77	23.8	Senior High School	Retirement	Urban	Spouse, Children	Family History of Cardiovascular Disease, Hypertension, Diabetes Mellitus, Hyperlipidemia	Coronary Artery Revascularization
P16	Male	50	27.7	Junior College	Employed	Rural	Spouse, Children	Hypertension, Hyperlipidemia	History of Myocardial Infarction, Angina Pectoris, Coronary Artery Revascularization
P17	Female	66	25.0	Junior High School	Retirement	Urban	Spouse	Family History of Cardiovascular Disease, Familial Hypercholesterolemia, Hyperlipidemia, Smoking	History of Myocardial Infarction, Coronary Artery Revascularization
P18	Male	63	26.9	Junior College	Retirement	Urban	Spouse, Children	Family History of Cardiovascular Disease, Familial Hypercholesterolemia, Hypertension, Diabetes Mellitus, Hyperlipidemia	History of Myocardial Infarction, Coronary Artery Revascularization
P19	Male	71	25.3	Senior High School	Retirement	Rural	Spouse, Children	Diabetes Mellitus	History of Myocardial Infarction, Coronary Artery Revascularization
P20	Male	72	25.8	Junior High School	Retirement	Urban	Spouse	Family History of Cardiovascular Disease, Hypertension, Diabetes Mellitus, Hyperlipidemia	Stroke
P21	Female	70	24.3	Primary School	Peasant	Rural	Spouse, Children	Hypertension, Diabetes Mellitus, Hyperlipidemia	/
P22	Female	65	24.9	Junior High School	Retirement	Urban	Spouse, Children	Family History of Cardiovascular Disease, Hypertension, Diabetes Mellitus, Hyperlipidemia	/

**Table 2:** Overview of Analyses and Examples.

Quotes	Subthemes	Themes
Q: What types of people are more likely to develop panvascular disease?		
“Long-term unhealthy habits such as smoking, alcohol consumption, a diet high in fatty meats and offal, and late-night sleep deprivation directly impair vascular health.”	Unhealthy Lifestyle Habits	Multifaceted Perceptions of Susceptibility
“With advancing age, weakened immunity and declining organ function create vulnerabilities, allowing unhealthy habits to take hold and diseases to develop.”	Age and Underlying Diseases	
“I have bad genes—my father had a stroke and family members have diabetes, so I’m likely affected too. “	Heredity and Family Aggregation	
“A poor mood and stress can affect blood pressure and emotional state, which is certainly detrimental to cardiovascular health.”	Stress and Emotion	
Q: If someone were to develop panvascular disease, would it be serious? What would be the consequences?		
“It could affect my hands or feet, or leave me partially paralyzed—any of that would make life difficult.”	Loss of Physical Function and Dignity	Multidimensional Fears of Severity
“If the illness is severe, a family member must stay and care for you, taking over your work and bearing an extra burden.”	Burden of Family Care	
“Caregivers, medications, check-ups, and rehabilitation all cost money.”	Economic Crisis	
“After falling ill, it’s nearly impossible to maintain an optimistic outlook for most.”	Psychological Burden and Lifestyle Restrictions	
Q: What do you see as the benefits of proactive screening?		
“Proactive screening helps us detect problems earlier and intervene in time, keeping blood lipids and pressure under control and avoiding surgery.”	Early Warning and Clinical Benefits	Positive Attitudes Toward the Benefits of Proactive Screening
“ It helps ease the worry about whether something might be wrong with your health.”	Psychological Security and Family Responsibility	
“Test results help target habit changes, like reducing greasy food. People tend to be lazy, so sometimes you need those indicators to drive change.”	Trigger for Health Behavior Change	
Q: What factors would hinder you from undergoing proactive screening?		
“I eat well and sleep well. I don’t have any issues, so I don’t need to get checked.”	Asymptomatic Neglect and Cognitive Blind Spots	Multiple Perceived Barriers to Proactive Screening
“Elderly individuals often lack a proper understanding of economic benefits, believing that spending money on medical care is not worthwhile and that it should not be wasted.”	Economic Burden and Value Trade-offs	
“Large hospitals have long queues—registering, seeing the doctor, paying, and testing all require waiting. “	Cumbersome and Inefficient Processes	
“Many elderly people find smartphones difficult. Registering and making appointments on phones is troublesome for them.”	Digital Divide and Technical Barriers	
“I usually avoid check-ups out of fear. Finding something wrong would weigh on my mind. Normal results relieve me; abnormal results keep me awake.”	Disease-Related Fear and Fatalistic Beliefs	
Q: Do you have confidence in conducting proactive screening? Why?		
“I’ve been through so many check-ups that I’m experienced now. I don’t worry anymore.”	Rich Experience Fosters Independent Confidence	Significant Differences in Self-Efficacy for Proactive Screening
“I searched online beforehand. Social media like WeChat and Xiaohongshu are helpful for learning about screening.”	Tools and Resources Enable Smooth Navigation	
“A family member should accompany me to hospital tests—it gives me peace of mind.”	High Dependency Undermines Action Confidence	

## Data Availability

The data presented in this study are not publicly available due to privacy or ethical restrictions.

## Supplementary Materials

The following supporting information can be downloaded at: https://www.mdpi.com/article/10.3390/healthcare14121766/s1, Supplementary File S1: interview outline.
