# Supplementary material for: Proactive Screening Beliefs in Chinese High-Risk Patients of Panvascular Disease from the Perspective of Health Belief Model: A Qualitative Study
Source: Healthcare (Basel). 2026 Jun 18;14(12):1766. doi: 10.3390/healthcare14121766 (PMC13300550; doi:10.3390/healthcare14121766)
Supplement: Supplementary file 1 [file healthcare-14-01766-s001.zip › healthcare-4284410-supplementary.pdf]

Supplementary File S1: interview outline

HBM Construct	Interview Question
	1. Have you heard of or do you know about panvascular disease? What do you think panvascular disease might refer to? Could you give some examples? (If they don't konw, explain to them the definition of panvascular disease without mentioning disease susceptibility or severity.)
Perceived susceptibility	2. what types of people are more likely to develop panvascular disease?
Perceived susceptibility	3. Do you think you are at risk of developing this disease? Why or why not?
Perceived severity	4. If someone were to develop panvascular disease, would it be serious? What would be the consequences?
	5. What tests have you undergone at the hospital before? This includes routine physical examinations and tests during medical visits.
	6. Which of these tests do you think are related to panvascular disease?
	7. In addition, what other screening methods are available for panvascular diseases?(If they don't konw, introduce them to the panvascular disease screening program.)
	8. Would you proactively undergo screening for panvascular disease?
Perceived benefits	9. If yes, why? What factors would prompt you to undergo proactive screening? In other words, what do you see as the benefits of proactive screening?
Perceived barriers	10. If no, why not? What factors would hinder you from undergoing proactive screening? In other words, what troubles or difficulties do you think proactive screening might bring you?
Self-efficacy	11. Do you have confidence in conducting proactive screening? Why?
